# Development of a Prediction Model for the Occurrence of Stenosis or Occlusion after Percutaneous Deep Venous Arterialization

**DOI:** 10.3390/diagnostics11061008

**Published:** 2021-05-31

**Authors:** Eline Huizing, Michiel A. Schreve, Steven Kum, Grigorios Papageorgiou, Jean-Paul P. M. de Vries, Gert J. de Borst, Çağdaş Ünlü

**Affiliations:** 1 Department of Surgery, Northwest Clinics, 1815 JD Alkmaar, The Netherlands; M.A.Schreve@nwz.nl (M.A.S.); Cagdas.Unlu@nwz.nl (Ç.Ü.); 2 Vascular Service, Department of Surgery, Changi General Hospital, Singapore 529889, Singapore; stevenkum.dr@gmail.com; 3 Department of Biostatistics, Erasmus University Medical Centre, 3015 GD Rotterdam, The Netherlands; g.papageorgiou@erasmusmc.nl; 4 Department of Epidemiology, Erasmus University Medical Centre, 3015 GD Rotterdam, The Netherlands; 5 Department of Surgery, Division of Vascular Surgery, University Medical Centre Groningen, 9713 GZ Groningen, The Netherlands; j.p.p.m.de.vries@umcg.nl; 6 Department of Vascular Surgery, University Medical Center Utrecht, 3584 CX Utrecht, The Netherlands; g.j.deborst-2@umcutrecht.nl

**Keywords:** venous arterialization, chronic limb threatening ischemia, duplex, restenosis, occlusion, reintervention, joint models, prediction, peak systolic velocity, volume flow, ultrasound

## Abstract

Percutaneous deep venous arterialization (pDVA) is a promising treatment option in patients with chronic limb-threatening ischemia. Stenosis and occlusions, which are the Achilles’ heel of every revascularization procedure, can be treated when detected early. However, frequent monitoring after pDVA is required because when stenosis or occlusions develop is unknown. Therefore, patients currently need to visit the hospital every 2 weeks for surveillance, which can be burdensome. Accordingly, we aimed to develop a model that can predict future stenosis or occlusions in patients after pDVA to be able to create tailor-made follow-up protocols. The data set included 343 peak systolic velocity and 335 volume flow measurements of 23 patients. A stenosis or occlusion developed in 17 patients, and 6 patients remained lesion-free. A statistically significant increase in the risk of stenosis or occlusion was found when duplex ultrasound values decreased 20% within 1 month. The prediction model was also able to estimate a patient-specific risk of future stenosis or occlusions. This is promising for the possibility of reducing the frequency of follow-up visits for low-risk patients and increasing the frequency for high-risk patients. These observations are the starting point for individual surveillance programs in post-pDVA patients. Future studies with a larger cohort are necessary for validation of this model.

## 1. Introduction

Percutaneous deep venous arterialization (pDVA) is a promising treatment option for patients with no-option chronic limb-threatening ischemia (CLTI) [1]. In the pDVA procedure, a fistula is made between an artery and vein, creating a new route for the oxygenated blood to reach the foot [2]. This new route is called the arteriovenous (AV) circuit and includes the inflow artery, crossing stent, stented vein, and outflow veins of the foot. 

After pDVA, the new AV circuit needs time to mature. In the first 6 weeks, the maturation consists of angiogenesis, which is seen in the forefoot. Stenosis or occlusion can occur during this period, limiting the patency of the AV construction [3,4]. Duplex ultrasound (DUS) scanning-derived peak systolic velocity (PSV) and volume flow (VF) can timely detect these obstructive lesions [3]. 

Currently, it is crucial for post-pDVA patients to visit the hospital every 2 weeks in the first 2 months after the procedure to monitor the AV circuit by DUS scanning [3]. This current demanding follow-up protocol is considered necessary to detect lesions that interfere with the maturation of the venous arterialization and therefore the success of wound healing. However, the frequent surveillance protocol is burdensome for these fragile patients and can be a possible hurdle to undergoing the pDVA procedure.

At present, there are no predictive indicators to decrease the number of follow-up visits while still being certain that significant stenoses will be detected in time. This limitation relies on the fact that the DUS values represent the actual patency. Progressive failure (i.e., future development of stenosis or occlusion) remains undetermined. 

A potential way to reduce these surveillance visits could be by identifying the risk for stenosis or occlusion in a certain time period. If the risk for stenosis or occlusion in, for example, 1 month could be estimated, it opens the opportunity to plan surveillance visits based on the estimated risk. 

An approach to estimate the future risk for stenosis or occlusions might be by evaluating a patient’s characteristics and DUS-measured values and by assessing the pattern that repeatedly measured DUS values show over time. However, whether there is a possibility to estimate the risk for stenosis or occlusion based on measured DUS values is yet unknown. We therefore investigated whether DUS-derived PSV and VF values can be used to build a prediction model for the occurrence of stenosis or occlusion. 

## 2. Materials and Methods

This study was conducted according to the principles of the Declaration of Helsinki and approved by the Institutional Board of Directors of Northwest Clinics, Alkmaar, The Netherlands, and Changi General Hospital, Singapore. The requirement for informed consent was waived because of the retrospective nature of the study.

### 2.1. Study Population

DUS measurements were retrospectively collected from patients who underwent a pDVA procedure from July 2014 to May 2020 in the Northwest Clinics in Alkmaar, The Netherlands, or Changi General Hospital in Singapore, Singapore. Patients with CLTI were eligible for the pDVA procedure. CLTI was defined as peripheral arterial disease in combination with rest pain, gangrene, or lower-limb ulceration longer than 2 weeks [5], in which no conventional revascularization procedure was deemed possible due to lack of a distal target artery, and with at least 1 patent tibial artery in the proximal segment. Exclusion criteria were acute limb ischemia, known deep vein thrombosis, extensive tissue loss that precluded limb salvage, and allergy or contraindication to antiplatelet and/or anticoagulation therapy [4].

The DUS measurements were performed from July 2014 to September 2020 and were collected retrospectively. Only values measured at the middle and distal segment of the stented vein were included for analysis because these had the highest combination of reliability and diagnostic accuracy for both the PSV and VF measurements to detect a failed AV circuit [3].

### 2.2. pDVA Procedure

The pDVA procedure was performed as described in detail previously [2,4]. In brief, arterial and venous accesses were created, and arterial and venous catheters were then inserted and advanced toward the crossing point. The connection was achieved by advancing a needle from the arterial catheter into the vein to create the arteriovenous fistula (AVF) using the LimFlow device (LimFlow SA, Paris, France) at the proximal part of the tibial vessels. A valvulotome was inserted to render the valves incompetent. Self-expanding stent grafts were implanted from the ankle upward to the crossing point. A tapered, covered self-expanding stent was used to secure the AVF and finalize the created AV circuit.

### 2.3. Follow-Up

After the procedure, patients were prescribed lifelong antiplatelet therapy along with anticoagulation for at least 3 months. Follow-up visits were planned every 2 weeks for the first 2 months and at 3, 6, and 12 months when possible. During these visits, DUS was used to assess the patency of the AV circuit, and the foot ulcer was evaluated. Patients were seen more frequently when abnormalities (e.g., absent flow, low PSV or VF values) were seen on DUS, if the ulcer deteriorated, if the patient had aberrant pain, or when reinterventions were performed. 

### 2.4. DUS Measurements

DUS measurements were performed as described previously [3]. In brief, measurements were performed using Affiniti 70G and IU22 (both from Philips, Amsterdam, The Netherlands) in The Netherlands and Singapore, respectively. 

The patient was examined supine with the hip of the measured leg rotated externally and the knee slightly flexed. An L12-3 linear array transducer was placed at the popliteal fossa in the transverse plane and moved distally. Grayscale and color Doppler imaging were used to check the vessel lumen and flow direction in transverse and longitudinal views for any abnormalities. The duplex Doppler mode was used to record PSV and VF measurements with the vessels in longitudinal views. A ≤ 60° Doppler angle with the cursor parallel to the vessel wall was used to measure the PSV. The sample volume was positioned in the center and completely encompassed the vessel lumen. On the Doppler trace, the baseline was lowered and the velocity scale was adjusted appropriately to avoid aliasing. To obtain the VF, the diameter of the vessel was measured with the calipers at right angles to the sample volume. Three pulse cycles on the spectral trace were selected, and the system automatically estimated the time-averaged mean and calculated the VF in mL/min. Surveillance of the AV circuit followed the same follow-up protocol as described above.

### 2.5. Data Collection and Study End Points 

The study cohort was previously described [3], except for 2 newly included patients. Data for those patients were collected retrospectively and included patient demographics, baseline risk factors, intraprocedural data, indications, reinterventions, and PSV and VF values. Data were derived from electronic medical records, clinical records, and imaging reports.

The primary outcome was to determine whether single DUS values or the pattern of repeatedly measured PSV and VF values over time can predict the occurrence of stenosis or occlusion in patients after pDVA. The secondary outcome was to assess whether a patient-specific risk of stenosis or occlusion can be estimated based on their measured PSV and VF values.

### 2.6. Statistical Analysis

Categorical variables are expressed as counts with percentage and were compared using the Fisher exact test. Continuous variables are presented as median and the interquartile range (IQR), and were compared using the Mann–Whitney *U* test.

Repeated measurements of PSV and VF values were analyzed using linear mixed-effects models. More specifically, for each of the different measurements, PSV mid, PSV distal, VF mid, and VF distal, a mixed-effects model was adjusted for age, sex, and reintervention at baseline. The logarithmic transformation of continuous dependent variables was used to satisfy the assumptions of normality and homoscedasticity. Natural cubic splines with 1 knot placed at the median value of time were used to allow for nonlinear evolution over time in both the fixed- and random-effects structure. Residual diagnostics, such as normal Q-Q plots and residuals vs. fitted values plots, were used to validate the assumptions of the models.

To further investigate the effect of reintervention as a time-varying variable, we considered an alternative modeling approach based on the assumption that using reintervention as a time-varying covariate might better explain changes in the longitudinal trajectories. Furthermore, it captures more accurately the process because reintervention occurred after baseline at different times for each patient. We achieved this by including time relative to reintervention as a time-varying covariate in the mixed-effects model, both as a fixed effect and as a random effect [6]. As a result of the added complexity and the relatively small sample size, linear effects of time were used in this modeling approach.

To complement the previous analyses and further explore the sensitivity of the underlying assumptions, we also considered a simpler approach in which repeated measurements were considered independent after a reintervention for stenosis or occlusion. The measurements that resulted were considered as a new series. For example, if 1 patient underwent 3 reinterventions during follow-up, the measurements between these reinterventions were considered as independent and separately analyzed. For this reason, the mixed-effects models were not adjusted for subject-specific covariates. Thus, 3 different models were developed.

**Figure 1:**
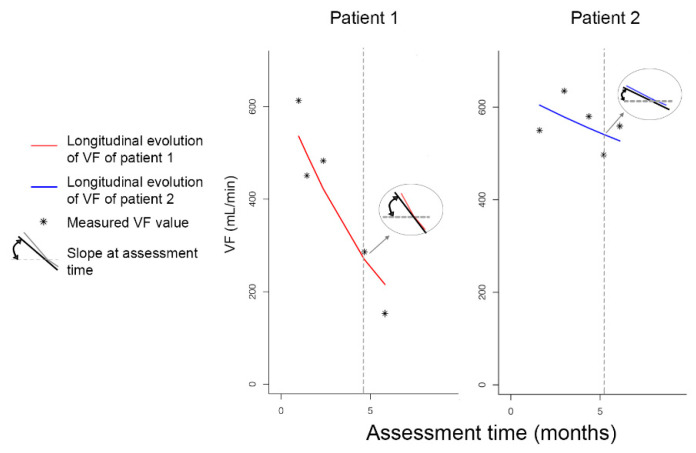
Illustration of a longitudinal evolution of patient 1, who eventually developed a stenosis or occlusion, and patient 2, who remained lesion-free. The figure illustrates the different parametrizations that can be assessed; that is, the DUS values and their pattern over time (i.e., the slope) of the values at assessment time.

A relative risk model was used to analyze the risk for stenosis or occlusion. To estimate the association between the repeatedly measured PSV and VF values and stenosis or occlusion, the estimated trajectories from the linear mixed-effects model were included in the relative risk model and were jointly analyzed under the joint modeling framework. Two association structures were considered: the current value association and the current slope association [7]. The former assumes that the risk for stenosis or occlusion at time *t* is associated with the value of the estimated longitudinal trajectory at the same time, whereas the latter assumes that the current rate of increase/decrease of the estimated longitudinal trajectory at time *t* is associated with the risk for stenosis or occlusion at the same time point. Results are presented as hazard ratios (HRs) with 95% confidence intervals (CIs) per 20% decrease in the PSV or VF value. The analyzed associations are illustrated in Figure 1.

All analyses were performed in R 4.0.3 software (R Foundation for Statistical Computing, Vienna, Austria), using the R packages JM [8], nlme, splines, and survival. Statistical significance was defined as *p* < 0.05.

## 3. Results

### 3.1. Patient Characteristics

The study included 23 patients who had DUS measurements at follow-up. A total of 7 patients were treated in The Netherlands and 16 in Singapore. Within a median follow-up of 6 months (IQR, 3.3–14.2 months), a stenosis or occlusion developed in 17 patients, and 6 remained lesion-free. There were 446 duplex investigations in the 17 patients with a stenosis/occlusion versus 232 investigations in the 6 lesion-free patients. Baseline characteristics are summarized in Table 1. Comorbidities included hypertension in 18 patients (79%), diabetes in 16 (70%), and hyperlipidemia in 17 (74%). There were 22 patients (95%) classified as Rutherford 5 or 6 and deemed at moderate or high risk of amputation according to the Society of Vascular Surgery Wound Ischemia Foot infection (WIfI) classification [9]. Statistically significantly more patients with hyperlipidemia were in the stenosis/occlusion group (*p* = 0.021). No other statistically significant differences were found between the groups.

### 3.2. PSV and VF Measurements

The data included 343 PSV measurements (173 mid stent measurements and 170 distal stent measurements) and 335 VF measurements (167 mid stent measurements and 168 distal stent measurements). The mean of the included consecutive measurements per patient per measuring point was 5. 

### 3.3. Association between Patients’ Characteristics and Duplex Values

Table A1, available in the Appendix A, represents the associations between patients’ baseline characteristics, time, and DUS values.

### 3.4. Pattern of Repeatedly Measured PSV and VF Values over Time

**Figure 2:**
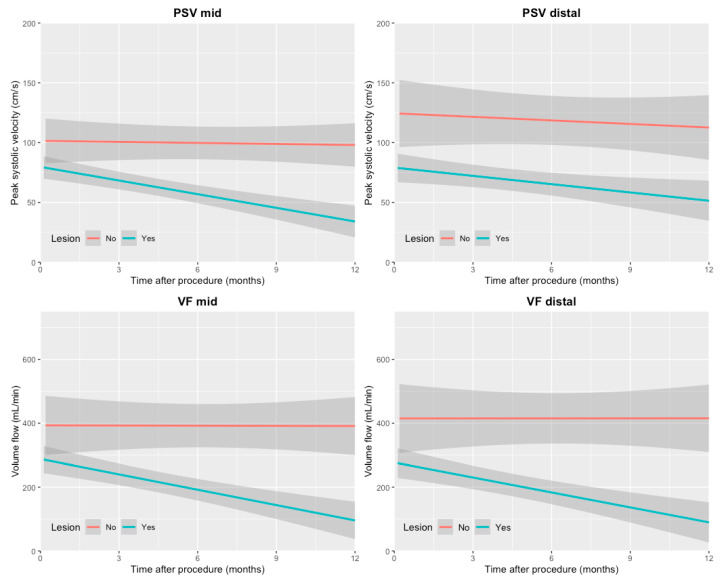
Illustration of the trends of repeatedly measured DUS values over time in patients with and without stenosis or occlusion. The shaded areas indicate the 95% confidence interval.

The pattern of repeatedly measured PSV and VF values over time showed a decreasing trend in patients with a stenosis or occlusion (Figure 2). In patients without stenosis or occlusion, the PSV and VF values were higher at baseline and remained more constant over time.

### 3.5. Association between Stenosis or Occlusion and Duplex Values

Of the 17 patients in whom primary patency was lost, 13 also lost secondary patency. In total, 47 restenosis and reocclusions occurred in the 17 patients, which were used for analyses.

#### 3.5.1. PSV Values

PSV values measured in the middle and distal parts of the stented vein were associated with the risk of stenosis or occlusion. All 3 different models showed a statistically significant association. The strongest association for the PSV value was found for the PSV measured in the mid-stent, with a decrease of 20% of PSV, resulting in a 1.5-fold (95% CI, 1.1–2.1) increase of the risk of stenosis or occlusion. 

The pattern of repeatedly measured DUS values over time was associated with the risk of stenosis or occlusion when estimated by the reintervention model and by the series model. The strongest association was found for the slope measured mid-stent, with a decrease of 20% in PSV value within 1 month corresponding to a 3.5-fold (95% CI, 1.6–7.8) increase in the risk. The results are summarized in Table 2.

#### 3.5.2. VF Values

The VF values measured mid and distal in the stent were also associated with the risk of stenosis or occlusion for the 3 models. The strongest association was found for the VF value measured mid-stent, with a 1.3-fold (95% CI, 1.1–1.5) increase in the risk of stenosis or occlusion for a 20% decrease in VF value. 

The pattern of repeatedly measured DUS values over time was only found associated when estimated by the basic model for the values measured mid-stent. A decrease of 20% in VF value within 1 month corresponded to a 5.0-fold (1.3–19.3) increase in the risk of stenosis or occlusion.

### 3.6. Patient-Specific Dynamic Prediction

**Figure 3:**
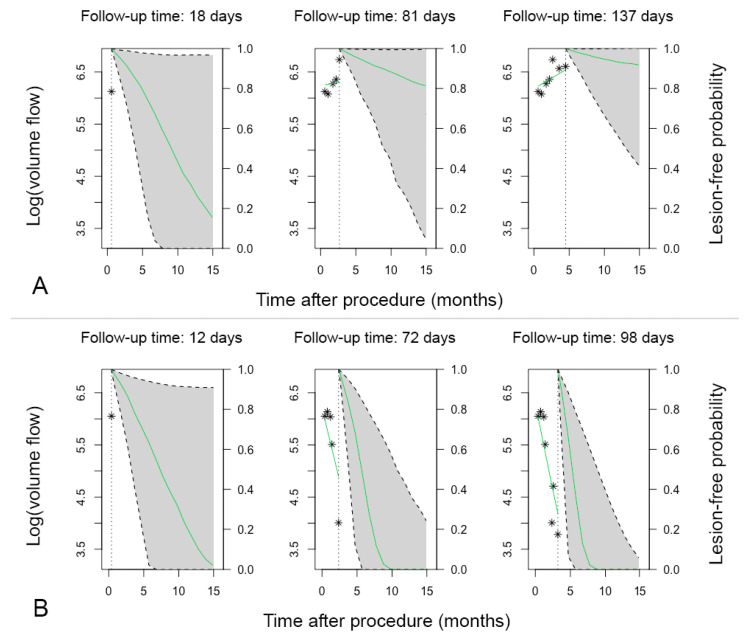
Illustration of a retrospectively estimated lesion-free probability by the developed model. The green line represents the hazard for stenosis or occlusion with corresponding 95% confident intervals in gray. The asterisks represent the measured VF values. (**A**) Illustration of the estimations of a patient who remained lesion-free after a reintervention at 58 days of follow-up. (**B**) In this patient, multiple stenoses were treated at 72 and 130 days of follow-up.

Figure 3 shows an example of 2 patients in whom we retrospectively estimated the lesion-free probability by their measured VF values using the developed reintervention model. In Figure 3A, a measurement was performed 18 days post procedure. The measured log VF was ±5.7 mL/min. The model estimated a lesion-free probability of 20% at 15 months post procedure. A clinically driven target lesion reintervention was performed 58 days post procedure because of a stenosis in the lateral plantar vein. The patient, who was effectively treated with a drug-coated balloon (DCB), was seen 81 days post procedure, and a log VF of ±6.7 mL/min was found. The corresponding lesion-free probability was updated according to the new measured values. The model estimated a lesion-free probability of 80% during follow-up. During the total 15 months of follow-up, no more clinically driven target lesions reinterventions were performed.

In Figure 3B, a patient was seen 12 days post procedure. The model estimated a lesion-free probability of 0% at 15 months post procedure according to the measured log VF value. The patient was seen again at 72 days with a decrease in the log VF value. The lesion-free probability was updated, and the model estimated a lesion-free probability of 0% at 10 months post procedure. A clinically driven target-lesion reintervention was performed due to a significant stenosis at the lateral plantar vein. The patient was effectively treated with a DCB. The VF values were measured at 98 days of follow-up, and the model estimated a corresponding lesion-free probability of 0% at 7 months post procedure. At 130 days post procedure, the patient underwent a reintervention for a restenosis of 50% at the lateral plantar vein. 

## 4. Discussion

In this study, we developed a prediction model to evaluate whether PSV and VF values can predict the occurrence of stenosis or occlusions over time as proof of concept. The possibility to estimate the risk of future stenosis or occlusion offers perspective for their use in surveillance of post-pDVA patients. In high-risk patients, the follow-up frequency can be increased to detect lesions before they develop into occlusions. In case of a low risk of stenosis or occlusions, the follow-up visit can be suspended or omitted. In this way, it is possible to allow high patency rates by early detection and minimal burden for the patient. This patient-based approach is desirable for this fragile patient population. However, the model needs to be validated first before it can be used in clinical practice.

The present study developed 3 models in which reinterventions were analyzed differently. In all models, we found substantial associations between DUS values and stenosis or occlusion. Our findings confirm that DUS measurements are clinically relevant for monitoring post-pDVA patients to timely detect stenosis and prevent occlusions. The strongest finding was found for PSV values measured mid-stent, with a 1.5-fold increase in the risk of stenosis or occlusion for a 20% decrease in PSV value. This could be helpful for physicians to better detect disease progression by the knowledge that there is an increased risk when a 20% decrease in PSV values is found. 

Even stronger effects were found when the trend of repeatedly measured VF values was analyzed. A 20% decrease in the VF value within 1 month corresponds to a 5.0-fold increase in the risk of stenosis of occlusion. This would imply that a sudden decrease reflects a present or upcoming patency loss, which is in line with our clinical experience. We would therefore suggest a thorough evaluation of the patient’s clinical status, including pain assessment, wound assessment, and transcutaneous oxygen pressure measurements, to make a well-considered decision for a possible angiography. 

The patient’s clinical assessment should always be considered, because the true strength of the association remains uncertain due to the small sample size of the study. Therefore, the presented hazard ratios should be interpreted with caution and only be used in consideration with the patient’s clinical status.

The present study used joint models under maximum likelihood to estimate the association between DUS values and the risk of stenosis or occlusion. Another alternative is to fit joint models under a Bayesian approach, which may be preferable because it provides greater flexibility for modeling and more capabilities to derive dynamic predictions [10]. However, these extensive options were beyond the scope of this proof-of-concept research. Furthermore, fitting models under a Bayesian approach for this study might be tricky, because the model always provides a result, even if the model does not converge properly. Therefore, for the present study, it was more appropriate to fit the model under maximum likelihood to obtain more reliable results.

Currently, DUS measurements are interpreted by using cutoff points [3], which are used for every patient. In the present study, we demonstrated that patient-specific risks could be calculated and that the risk was updated after every new measurement. This approach can possibly be used for any other vascular reconstructions and can potentially lead to a personalized patient follow-up, which can change the general vascular follow-up practice dramatically.

To facilitate calculating patient-specific predictions, a web interface can be used. The package “JMbayes” for R facilitates use for this [10]. The ability to calculate a patient-specific risk by entering DUS measured values on a website simplifies its use in practice. A physician can easily enter the values, and the risk will be displayed on screen, similar to Figure 3. This makes the use of joint models for clinical practice easily accessible. 

Because this is a proof-of–concept study, limitations are inevitable. A substantial drawback is the small group size, and the results obtained may only reflect the included population and might be unreliable when extrapolated to a larger population. Owing to the small sample size, the predictive accuracy, prediction error, and validation of the models could not be specifically determined. A larger sample size is desirable to validate the prediction model; however, the pDVA procedure is only performed in patients with no-option CLTI, which is a relatively small population.

Acknowledging the limitations of the study, the present study could still contribute to interpreting DUS values in post-pDVA patients because the current knowledge of DUS measurements in post-pDVA patients is limited. Nevertheless, the accuracy, validation, and reliability of the model should be analyzed in future studies based on a larger sample size. 

## 5. Conclusions

Our preliminary data suggest that DUS values can predict the occurrence of stenosis and occlusion and that a prediction model can be made. Our observations need to be confirmed in a larger cohort before firm conclusions can be drawn.

## Figures and Tables

**Table 1:** Baseline characteristics of included patients.

	Total	Stenosis/Occlusion		
Variable		No	Yes	*p* Value
Patients	23	6 (26)	17 (74)	
Men	9 (40)	4 (67)	5 (29)	0.162
Age, years	63 (56–82)	55 (35–73)	72 (58–85)	0.107
Comorbidities				
Hypertension	18 (79)	3 (50)	15 (88)	0.089
Diabetes	16 (70)	3 (50)	13 (77)	0.318
Hyperlipidemia	17 (74)	2 (33)	15 (88)	0.021
Cerebrovascular accident	4 (17)	0 (0)	4 (24)	0.539
Coronary artery disease	7 (30)	1 (17)	6 (35)	0.621
Dialysis dependent	2 (9)	1 (27)	1 (6)	0.462
Body mass index, kg/m^2^	23 (19–25)	24 (22–26)	21 (19–24)	0.100
Laboratory results				
Creatinine, mg/dL	86 (66–145)	108 (82–179)	84 (65–152)	0.302
eGFR <30 mL/min/1.73 m^2^	5 (24)	1 (20)	4 (25)	1.000
Rutherford				
4	1 (4)	0 (0)	1 (6)	1.000
5	15 (65)	5 (83)	10 (59)	0.369
6	7 (30)	1 (17)	6 (35)	0.621
SVS WIfI risk staging				
Low risk	1 (4)	0 (0)	1 (6)	1.000
Moderate risk	5 (22)	1 (17)	4 (24)	1.000
High risk	17 (74)	5 (83)	12 (71)	1.000

Categorical variables are presented as *n* (%) and continuous variables as median (interquartile range). eGFR = glomerular filtration rate, SVS WIfI = Society for Vascular Surgery risk system based on Wound, Ischemia and foot Infection.

**Table 2:** Association between duplex values and stenosis or occlusion as estimated by the three different models.

	PSV Mid		PSV Distal		VF Mid		VF Distal	
	HR (95% CI)	*p* Value	HR (95% CI)	*p* Value	HR (95% CI)	*p* Value	HR (95% CI)	*p* Value
Basic model								
Value	1.34 (1.05–1.71)	0.018	1.37 (1.14–1.65)	0.001	1.15 (1.04–1.28)	0.007	1.15 (1.01–1.31)	0.032
Slope	1.57 (0.75–3.29)	0.229	1.10 (0.93–1.31)	0.276	5.02 (1.31–19.32)	0.019	1.08 (0.62–1.88)	0.790
Reintervention model								
Value	1.51 (1.12–2.05)	0.008	1.34 (1.04–1.72)	0.024	1.28 (1.07–1.54)	0.008	1.23 (1.05–1.45)	0.012
Slope	2.76 (1.09–7.02)	0.033	1.66 (0.71–3.89)	0.243	3.18 (0.97–10.38)	0.056	3.06 (0.97–9.63)	0.056
Per series model								
Value	1.32 (1.17–1.50)	<0.001	1.36 (1.19–1.56)	<0.001	1.11 (1.03–1.21)	0.009	1.22 (1.06–1.14)	<0.001
Slope	3.49 (1.56–7.80)	0.002	1.30 (1.06–1.58)	0.010	0.95 (0.54–1.65)	0.845	1.22 (0.52–2.86)	0.642

Results are shown per 20% decrease of the measured value and per 20% decrease of the measured value within a month (slope term). HR = hazard ratio, CI = confidence interval.

## Appendix A

**Table A1:** Association between baseline characteristics and serially measured PSV and VF values.

Model			PSV Mid		PSV Distal		VF Mid		VF Distal	
Basic			Est. (95% CI)	*p* Value	Est. (95% CI)	*p* Value	Est. (95% CI)	*p* Value	Est. (95% CI)	*p* Value
	Value	Time 1	0.96 (0.91–1.02)	0.214	0.13 (0.09–0.19)	<0.0001	0.85 (0.80–0.91)	<0.0001	0.08 (0.02–0.30)	<0.001
		Time 2			0.32 (0.25–0.42)	<0.0001			0.93 (0.07–11.94)	0.953
		Age	0.98 (0.97–0.99)	0.005	0.98 (0.98–0.99)	<0.0001	0.99 (0.98–1.00)	0.1007	0.98 (0.96–0.99)	0.008
		Female sex	0.71 (0.53–0.96)	0.028	0.66 (0.57–0.78)	<0.0001	0.71 (0.51–0.99)	0.0437	0.53 (0.16–1.74)	0.295
		Reintervention	0.98 (0.58–1.64)	0.926	1.09 (0.92–1.31)	0.314	0.84 (0.57–1.23)	0.3724	1.12 (0.38–3.25)	0.837
	Slope	Time 1	0.96 (0.92–1.01)	0.103	0.20 (0.13–0.31)	<0.0001	0.84 (0.81–0.87)	<0.0001	0.05 (0.03–0.09)	<0.0001
		Time 2			0.48 (0.33–0.69)	<0.001			0.69 (0.53–0.90)	0.007
		Age	0.98 (0.97–0.99)	0.003	0.98 (0.97–0.99)	<0.0001	0.99 (0.98–1.01)	0.2517	0.98 (0.97–0.98)	<0.0001
		Female sex	0.71 (0.53–0.95)	0.023	0.67 (0.53–0.85)	0.001	0.73 (0.53–1.01)	0.0553	0.47 (0.37–0.60)	<0.0001
		Reintervention	0.94 (0.63–1.40)	0.772	1.15 (0.90–1.47)	0.274	0.76 (0.51–1.13)	0.1751	1.34 (0.97–1.84)	0.077
Reintervention	Value	Time 1	0.10 (0.04–2.53)	<0.0001	0.14 (0.05–0.38)	<0.001	0.80 (0.76–0.85)	<0.0001	0.78 (0.72–0.84)	<0.0001
		Time 2	0.54 (0.29–1.01)	0.053	1.71 (0.85–3.41)	0.130				
		Time relative	1.01 (0.97–1.06)	0.645	1.26 (0.92–1.71)	0.147	1.05 (0.96–1.14)	0.3294	1.09 (0.99–1.20)	0.089
		Age	0.99 (0.98–1.00)	0.011	0.98 (0.97–0.99)	0.001	0.99 (0.97–1.00)	0.0411	0.98 (0.97–1.00)	0.010
		Time × time relative			0.73 (0.44–1.20)	0.211				
		Time × time relative			0.82 (0.66–1.03)	0.089				
	Slope	Time 1	0.11 (0.05–0.25)	<0.0001	0.14 (0.05–0.41)	<0.001	0.79 (0.76–0.82)	<0.0001	0.76 (0.73–0.80)	<0.0001
		Time 2	0.47 (0.29–0.77)	0.003	1.69 (0.84–3.39)	0.141				
		Time relative	1.02 (0.98–1.07)	0.315	1.30 (0.94–1.79)	0.114	1.09 (1.00–1.18)	0.0591	1.13 (1.04–1.24)	0.007
		Age	0.99 (0.98–1.00)	0.002	0.98 (0.97–0.99)	0.001	0.99 (0.98–1.00)	0.0383	0.98 (0.97–1.00)	0.009
		Time × time relative			0.71 (0.42–1.20)	0.201				
		Time × time relative			0.80 (0.64–1.01)	0.057				
Series	Value	Time 1	0.74 (0.73–0.76)	<0.0001	0.07 (0.04–0.14)	<0.0001	0.10 (0.04–0.28)	<0.0001	0.05 (0.02–0.09)	<0.0001
		Time 2			0.59 (0.43–0.81)	0.001	0.23 (0.07–0.75)	0.0148	0.26 (0.21–0.33)	<0.0001
	Slope	Time 1	0.74 (0.73–0.76)	<0.0001	0.09 (0.06–0.13)	<0.0001	0.14 (0.09–0.22)	<0.0001	0.16 (0.10–0.25)	<0.0001
		Time 2			0.57 (0.48–0.68)	<0.0001	0.31 (0.24–0.41)	<0.0001	0.39 (0.30–0.51)	<0.0001

CI = confidence interval.
